# WMJ-S-001, a novel aliphatic hydroxamate derivative, exhibits anti-angiogenic activities via Src-homology-2-domain-containing protein tyrosine phosphatase 1

**DOI:** 10.18632/oncotarget.2765

**Published:** 2014-11-16

**Authors:** Yi-Fang Chang, Ya-Fen Hsu, Pei-Ting Chiu, Wei-Jan Huang, Shiu-Wen Huang, George Ou, Joen-Rong Sheu, Ming-Jen Hsu

**Affiliations:** ^1^ Graduate Institute of Clinical Medicine, College of Medicine, Taipei Medical University, Taipei, Taiwan; ^2^ Division of Hematology and Oncology, Department of Internal Medicine, MacKay Memorial Hospital, Taipei, Taiwan; ^3^ Division of General Surgery, Department of Surgery, Landseed Hospital, Taoyuan, Taiwan; ^4^ Graduate Institute of Medical Sciences, College of Medicine, Taipei Medical University, Taipei, Taiwan; ^5^ Graduate Institute of Pharmacognosy, Taipei Medical University, Taipei, Taiwan; ^6^ Graduate Institute of Pharmacology, College of Medicine, National Taiwan University, Taipei, Taiwan; ^7^ Department of Medicine, University of British Columbia, Vancouver, British Columbia, Canada; ^8^ Department of Pharmacology, School of Medicine, College of Medicine, Taipei Medical University, Taipei, Taiwan

**Keywords:** Angiogenesis, Endothelial cells, Hydroxamate, p53, VEGF

## Abstract

Angiogenesis, one of the major routes for tumor invasion and metastasis represents a rational target for therapeutic intervention. Recent development in drug discovery has highlighted the diverse biological and pharmacological properties of hydroxamate derivatives. In this study, we characterized the anti-angiogenic activities of a novel aliphatic hydroxamate, WMJ-S-001, in an effort to develop novel angiogenesis inhibitors. WMJ-S-001 inhibited vascular endothelial growth factor (VEGF)-A-induced proliferation, invasion and endothelial tube formation of human umbilical endothelial cells (HUVECs). WMJ-S-001 suppressed VEGF-A-induced microvessel sprouting from aortic rings, and attenuated angiogenesis in *in vivo* mouse xenograft models. In addition, WMJ-S-001 inhibited the phosphorylations of VEGFR2, Src, FAK, Akt and ERK in VEGF-A-stimulated HUVECs. WMJ-S-001 caused an increase in SHP-1 phosphatase activity, whereas NSC-87877, a SHP-1 inhibitor, restored WMJ-S-001 suppression of VEGFR2 phosphorylation and cell proliferation. Furthermore, WMJ-S-001 inhibited cell cycle progression and induced cell apoptosis in HUVECs. These results are associated with p53 phosphorylation and acetylation and the modulation of p21 and survivin. Taken together, WMJ-S-001 was shown to modulate vascular endothelial cell remodeling through inhibiting VEGFR2 signaling and induction of apoptosis. These results also support the role of WMJ-S-001 as a potential drug candidate and warrant the clinical development in the treatment of cancer.

## INTRODUCTION

Angiogenesis is a complex process of forming new blood vessels from preexisting vasculature [1]. It occurs primarily in many physiological processes such as embryogenesis, menstruation and wound healing, but it is also implicated in various pathological events including arthritis, psoriasis, diabetic retinopathy and cancer [2]. Angiogenesis is a key step for tumor progression and metastasis. Tumor vascularity also usually correlates with poor outcome. Modulating tumor-associated angiogenesis thus represents a promising strategy for the development of anti-cancer therapies [3, 4]. Angiogenesis is regulated by the balance between angiogenic and anti-angiogenic signaling. Although oncogenic events may enhance tumor cell survival during the initial avascular phase, subsequent large-scale tumor growth requires blood supply. The balance between angiogenic and anti-angiogenic signaling therefore shifts towards blood vessel formation to ensure adequate blood supply for tumor cell growth [5]. This “angiogenic switch” initiates angiogenesis which requires integrated actions of a number of angiogenic factors [6]. Numerous angiogenic factors including vascular endothelial growth factor (VEGF) [7], epidermal growth factor (EGF), basic fibroblast growth factor (bFGF) [8, 9] and angiopoietin [10] have been implicated in tumor angiogenesis. Of these, VEGF-A, a member of the VEGF family, plays a pivotal role in tumor angiogenesis [11, 12]. VEGF-A is over-expressed in a variety of tumors [13],and augments most steps of angiogenesis such as increasing vascular permeability and endothelial cell proliferation, migration and invasion into the surrounding tissue [14]. Cellular responses to VEGF-A are mainly mediated by the receptor tyrosine kinase VEGFR2 (also known as Flk-1) on the surface of endothelial cells [15]. Activation of VEGFR2 turns on the signaling cascades including Src family kinases, Akt (also known as protein kinase B), focal adhesion kinase (FAK), and extracellular signal-regulated kinase (ERK) [16]. These signaling cascades are involved in the regulation of endothelial cell proliferation, migration and survival [17-19]. In addition, VEGFR2 associates with SH2 domain-containing protein tyrosine phosphatase-1 (SHP-1) [20], the silencing of which accelerates angiogenesis in a rat model [21]. It suggests that tyrosine phosphatase-mediated inactivation may provide another means of regulating VEGFR2 signaling. Thus VEGF-VEGFR2 signaling represents an attractive target for anti-angiogenesis therapy of cancer [22]. Various strategies to interfere with VEGF-A-VEGFR signaling are currently being assessed in clinical trials. These include soluble receptors that sequester VEGF-A [23], antibodies targeting VEGF-A or VEGFR [24], and small molecule inhibitors of VEGFR2 [25]. To date, monoclonal antibody such as bevacizumab [26] and small molecule inhibitors such as sorafenib and sunitinib have already been approved by the United States Food and Drug Administration for treating certain tumor types [27].

Recent development in drug discovery has highlighted the diverse biological and pharmacological properties of hydroxamate, a key pharmacophore [28]. A variety of hydroxamate derivatives have demonstrated their potential use as anti-inflammatory [29], anti-infectious [30], or anti-tumor [31, 32] agents. They have also been shown to exhibit anti-angiogenic properties [33], but the mechanisms for this effect remain unclear at this time. Given their potential as lead compound for drug discovery, we synthesized a series of aliphatic hydroxamate derivatives, the WMJ-S compounds, and evaluated their anti-angiogenic properties. We also examined SHP-1's role in WMJ-S compounds’ anti-angiogenic actions.

**Fig.1:**
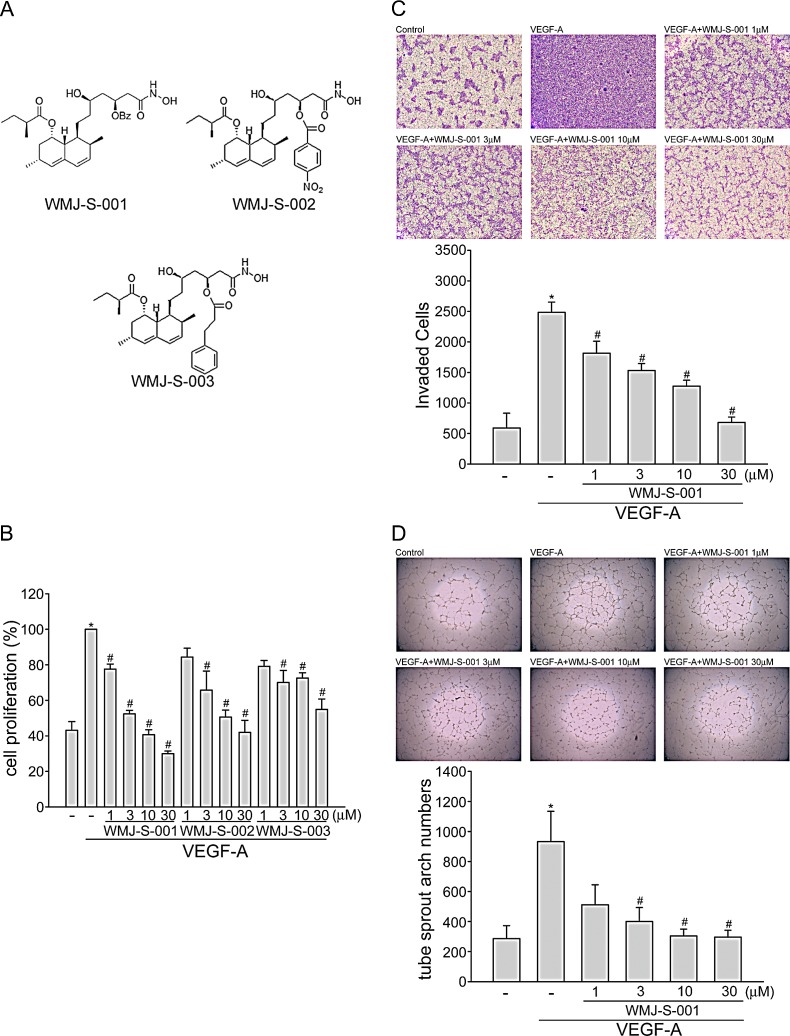
WMJ-S-001 inhibited VEGF-A-induced cell proliferation, invasion and tube formation (A) Chemical structures of WMJ-S-001, WMJ-S-002 and WMJ-S-003 (B) HUVECs were starved in 2 % FBS containing medium without ECGS for 16 h. After starvation, cells were pretreated with indicated concentrations of WMJ-S-001, WMJ-S-002 or WMJ-S-003 followed by the stimulation with VEGF-A (20 ng/ml) for another 24 h. Cell proliferation was determined as described in the *Materials and Methods* section. Each column represents the mean ± SEM of three independent experiments performed in duplicate. *p < 0.05, compared with the group treated with VEGF alone. (C) After starvation as described in (B), cells were then seeded in the top chamber in the absence or presence of WMJ-S-001 at indicated concentrations using VEGF-A as chemo-attractant. After 16 h, invaded cells through the gelatin-coated membrane were stained and quantified. Each column represents the mean ± S.E.M. of four independent experiments. *p < 0.05, compared with the group treated with VEGF-A alone. (D) HUVECs were seeded on Matrigel in the presence of VEGF-A (20 ng/ml) with or without WMJ-S-001 at indicated concentrations. Cells were then photographed under phase-contrast after 16 h. Bar graphs show compiled data of average sprout arch numbers (n=4). *p < 0.05, compared with the group treated with VEGF-A alone.

WMJ-S-001 (Fig. 1A) was selected among this group of compounds displaying potent inhibitory activities in VEGF-A-stimulated human umbilical endothelial cells (HUVECs). In this study, WMJ-S-001 activated SHP-1 to suppress VEGFR2 signaling and subsequent angiogenesis in VEGF-A-stimulated HUVECs. WMJ-S-001 also activated p53 signaling, leading to HUVEC apoptosis. Similarly, WMJ-S-001 suppressed angiogenesis in VEGF-A- and HCT116 colorectal cancer cells-induced angiogenesis xenograft model. Taken together, these results suggest the potential of WMJ-S-001 as a therapeutic agent with activity against tumor angiogenesis.

## RESULTS

### WMJ-S-001 inhibits VEGF-A-induced cell proliferation, migration and tube formation of HUVECs

Angiogenesis is a complex process that involves several discrete steps including endothelial cell proliferation, migration, invasion, and tube formation [34]. To investigate whether aliphatic hydroxamate derivatives, the WMJ-S compounds (Fig.1A), exhibit anti-angiogenic activities, BrdU labeling analysis was employed to determine the effects of WMJ-S-001, WMJ-S-002 and WMJ-S-003 on cell proliferation in VEGF-A-stimulated HUVECs. Cells were starved with 2 % FBS-containing medium for 16 h, and then stimulated by VEGF-A (20 ng/ml) in the presence or absence of the WMJ-S compounds (1-30 μM) for 24 h. As shown in Fig. 1B, the percentage of BrdU-labeled cells significantly increased after a 24 h VEGF-A treatment as compared with the vehicle-treated group, but this increase was reversed by WMJ-S-001, WMJ-S-002 or WMJ-S-003 in a concentration-dependent manner (Fig. 1B). Because WMJ-S-001 exhibited the most marked inhibitory effects, we sought to further investigate its inhibitory mechanisms in VEGF-A-stimulated HUVECs. We next used transwell invasion assay to determine the effect of WMJ-S-001 on HUVEC invasion, a pivotal step in angiogenesis [35]. As shown in Fig. 1C, using VEGF-A as the chemoattractant, WMJ-S-001 significantly reduced the number of invaded cells through the gelatin-coated transwell membrane barrier (Fig. 1C). Tubular formation of endothelial cells is also a key step in angiogenesis. To assess WMJ-S-001's effect on this step, HUVECs seeded on the surface of Matrigel in the presence of VEGF-A were treated with either WMJ-S-001 or vehicle as control. As shown in Fig. 1D, cells treated with VEGF-A became elongated, and formed capillary-like structure within 16 h. However, the addition of WMJ-S-001 concentration-dependently suppressed the formation of capillary-like network (Fig. 1D). These results indicate that WMJ-S-001 exerts anti-angiogenic activity through inhibition of cell proliferation, invasion and tube formation of endothelial cells.

### WMJ-S-001 suppresses VEGF-A-induced microvessel sprouting ex vivo, and tumor cells-induced angiogenesis *in vivo*

**Fig.2:**
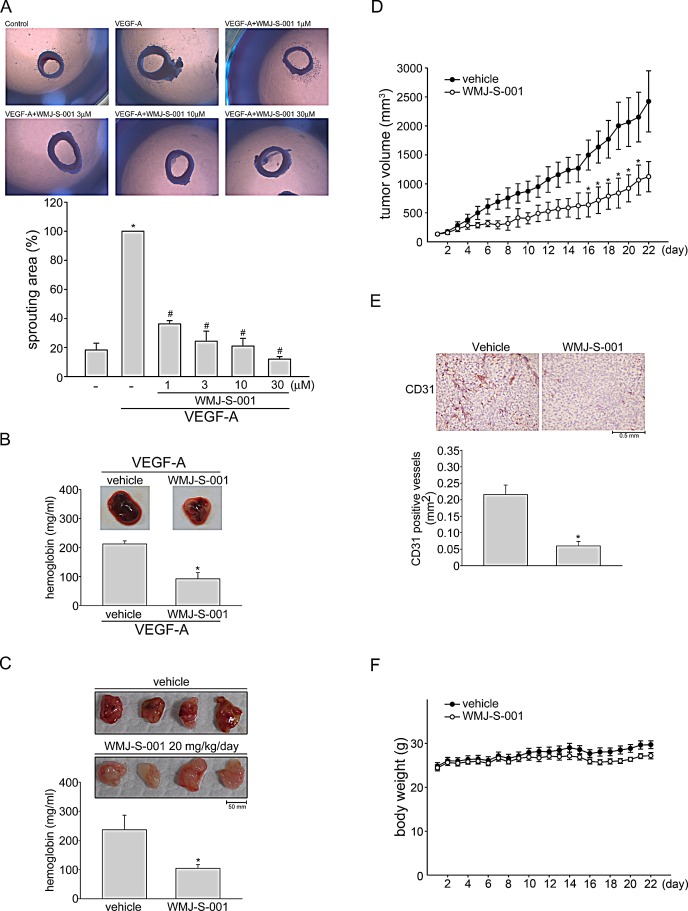
WMJ-S-001 inhibited angiogenesis and tumor growth in a mouse xenograft model Rat aortic rings were placed in Matrigel and treated with VEGF-A (20 ng/ml) in the presence or absence of WMJ-S-001 at indicated concentrations. The effect of WMJ-S-001 on formation of vessel sprout from various aorta samples was examined on day 8. Bar graphs show compiled data of average microvessels area (n=4). *p < 0.05, compared with the group treated with VEGF-A alone. (B) Matrigel mixed with VEGF-A was injected subcutaneously into the right flank of nude mice. After implantation, animals were treated intraperitoneally with vehicle or WMJ-S-001 (20 mg/kg/day) for 10 days. Matrigel plugs removed from the mice administered intraperitoneally with vehicle or WMJ-S-001 were shown. Hemoglobin levels in the Matrigel plug were quantified. Each column represents the mean ± S.E.M. of four plugs in each group (^*^p < 0.05 as compared with the vehicle-treated control group, n = 4). (C) HCT 116 colorectal cancer cells were mixed with Matrigel and then injected subcutaneously into the right flank of nude mice. After implantation, animals were treated intraperitoneally with vehicle or WMJ-S-001 (20 mg/kg/day) for 10 days. Matrigel plugs removed from the mice administered intraperitoneally with vehicle or WMJ-S-001 were shown. Hemoglobin levels in the Matrigel plug were quantified. Each column represents the mean ± S.E.M. of six plugs in each group (^*^p < 0.05 as compared with the vehicle-treated control group, n = 6). (D) Nude mice bearing xenografts of HCT116 cells were treated intraperitoneally with vehicle or WMJ-S-001 (20 mg/kg/day) for 22 days. The control group received vehicle only. Tumor volumes were calculated as described in the *Materials and Methods* section. Values represents the mean ± S.E.M. (^*^p < 0.05 as compared with the vehicle-treated control group, n = 5). (E) After 22 days of treatment, mice were sacrificed and the blood vessels in solid tumor sections were stained with anti-CD31 antibody. Images of immunohistochemical staining representative of eight randomly selected xenograft tumors (four tumors from each group) with similar results are shown. Compiled results are shown at the bottom of the chart. Each column represents the mean ± S.E.M. of four tumors from each group (^*^p < 0.05 as compared with the vehicle-treated control group). (F) No significant differences in body weights were found between the vehicle- and WMJ-S-001-treated groups.

We next explored the effects of WMJ-S-001 on VEGF-A-induced angiogenesis using an ex vivo rat aortic ring microvessel sprouting assay. As shown in Fig. 2A, VEGF-A triggered microvessel sprouting, leading to the formation of a complex network of microvessels around the aortic rings. Treatment with WMJ-S-001 markedly inhibited this phenomenon (Fig. 2A). We used VEGF-A-induced angiogenesis Matrigel plug assay to study the anti-angiogenic effects of WMJ-S-001 *in vivo*. As shown in Fig. 2B, significant blood vessel formation was observed in VEGF-A-supplemented Matrigel plug. The pale color of the plugs removed from the mice treated with WMJ-S-001 (20 mg/kg/day) indicates that VEGF-A induced less neovascularization after 10-day treatment with WMJ-S-001 (Fig. 2B, upper panel). The level of angiogenesis was quantified by determining the hemoglobin content of the plugs. A marked reduction in neovascularization was shown in plugs from WMJ-S-001-treated mice when compared with those from vehicle-treated mice (Fig. 2B, bottom panel). We also used a tumor cell-induced angiogenesis model to investigate WMJ-S-001's anti-angiogenic effects. HCT116 colorectal cancer cells were mixed with Matrigel and injected into the flanks of mice. Matrigel plugs were harvested 10 days after implantation. As shown in Fig. 2C, HCT116 cells induced blood vessel formation in the plug (Fig. 2C, upper panel). In contrast, the pale color of the plugs removed from the mice treated with WMJ-S-001 (20 mg/kg/day) indicates that HCT116 cells’ angiogenic effect was mitigated (Fig. 2C, upper panel). We also quantified the level of angiogenesis by determining the hemoglobin content of the plugs. A marked reduction in neovascularization was shown in plugs from WMJ-S-001-treated mice when compared with those from vehicle-treated control mice (Fig. 2C, bottom panel). These data indicate that systemic administration with WMJ-S-001 significantly suppressed angiogenesis in this *in vivo* assay.

### WMJ-S-001 suppressed colorectal tumor growth in a mouse xenograft model

We also used a mouse xenograft colorectal tumor model to investigate the effects of WMJ-S-001 on tumor growth. HCT116 colorectal cancer cells were injected into the flanks of mice. After allowing the tumors to grow subcutaneously to an average size of about 150 mm^3^, animals were treated with either vehicle or WMJ-S-001 (20 mg/kg/day) by daily intraperitoneal injections (I.P.) for 22 days. At the end of 22 days, mice were sacrificed and tissue samples were collected. As shown in Fig. 2D, WMJ-S-001 markedly reduced tumor growth comparing to the vehicle-treated control group. To further investigate whether WMJ-S-001 inhibits tumor angiogenesis, we used an anti-CD31 antibody to stain sections of the solid tumors. As shown in Fig. 2E, the tumor blood vessels in WMJ-S-001-treated tumors were clearly fewer than in the sections from the vehicle-treated control group. These results indicate that WMJ-S-001 inhibits tumor growth through, at least in part, suppressing tumor angiogenesis. In addition, no significant differences in body weights were found among the vehicle- and WMJ-S-001-treated groups throughout the whole experiment (Fig. 2F).

### WMJ-S-001 suppresses VEGF-A-induced Src, FAK, Akt and ERK phosphorylation

**Fig.3:**
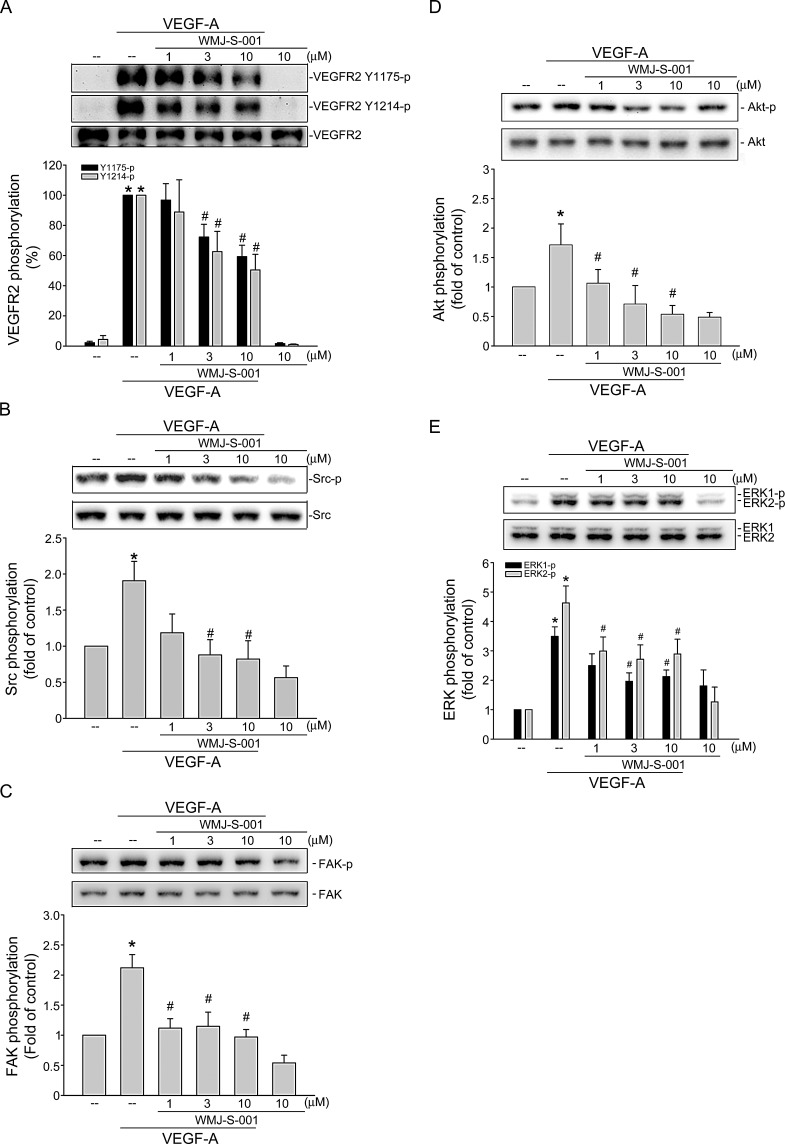
WMJ-S-001 inhibited VEGFR2 signaling pathways in HUVECs Cells were pretreated with indicated concentrations of WMJ-S-001 for 30 min, followed by the addition with VEGF-A (20 ng/ml) for another 5 (VEGFR2) or 20 (Src, FAK, Akt and ERK1/2) min. Phosphorylation status of VEGFR2 (A), Src (B), FAK (C), Akt (D), and ERK1/2 (E) were then determined by immunoblotting. The compiled results of VEGFR2 Tyr1175 and Tyr1214 (A), Src Tyr416 (B), FAK Tyr397 (C), Akt Ser473 (D), and ERK1/2 Thr202/Tyr204 (E) phosphorylations are shown. Each column represents the mean ± S.E.M. of at least four independent experiments. *p < 0.05, compared with the control group; #*p* < 0.05, compared with the group treated with VEGF-A alone.

VEGF-A signaling via VEGFR2 is the most important pathway in inducing angiogenesis [36]. There are several tyrosine residues on VEGFR2 that become phosphorylated upon VEGF-A exposure. Among these, tyrosine residues 1175 and 1214 are the two major VEGF-A-dependent autophosphorylation sites of VEGFR2 [37]. We therefore sought to determine whether WMJ-S-001 affects VEGFR2 Tyr1175 and Tyr 1214 phosphorylation in HUVECs after VEGF-A exposure. We also examined the phosphorylation status of Src, FAK, Akt and ERK, which are the essential protein kinases downstream of VEGFR2 signaling. As shown in Fig. 3A, WMJ-S-001 inhibited VEGFR2 Tyr1175 and Tyr 1214 phosphorylation in VEGF-stimulated HUVECs (Fig. 3A). WMJ-S-001 also suppressed the phosphorylation of Src (Fig. 3B), FAK (Fig. 3C), Akt (Fig. 3D) and ERK (Fig. 3E) in VEGF-stimulated HUVECs. Together, these results suggest that WMJ-S-001 exerts its anti-angiogenic activities by inhibiting VEGFR2 signaling.

### SHP-1 contributes to WMJ-S-001's inhibitory actions in VEGF-A-stimulated HUVECs

**Fig.4:**
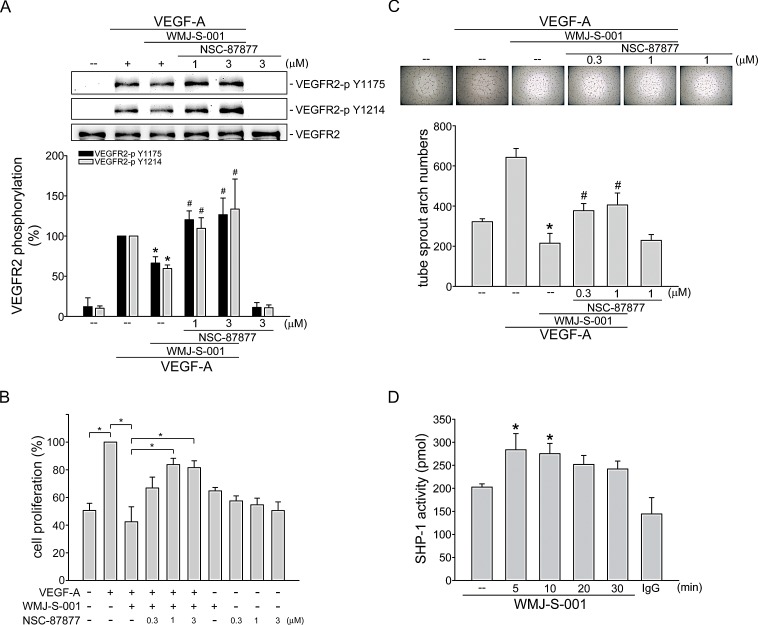
SHP-1 in WMJ-S-001 suppression of VEGF-A-induced VEGFR2 phosphorylation, cell proliferation and tube formation HUVECs were treated with SHP-1 inhibitor NSC-87877 for 30 min, followed by the addition with WMJ-S-001 (10 μM) for another 30 min. After treatment, cells were treated with VEGF-A (20 ng/ml) for another 5 min. Phosphorylation status of VEGFR2 was then determined by immunoblotting. The compiled results of VEGFR2 Tyr1175 and Tyr1214 phosphorylations are shown. Each column represents the mean ± S.E.M. of at least five independent experiments. *p < 0.05, compared with the control group; #*p* < 0.05, compared with the group treated with VEGF-A alone. (B) HUVECs were starved for 16 h. After starvation, cells were treated with SHP-1 inhibitor NSC-87877 for 30 min, followed by the addition with WMJ-S-001 (10 μM) for another 30 min. After treatment, cells were stimulated with VEGF-A (20 ng/ml) for another 24 h. Cell proliferation was determined as described in the *Materials and Methods* section. Each column represents the mean ± S.E.M. of at least six independent experiments. *p < 0.05, compared with the control group; #*p* < 0.05, compared with the group treated with VEGF-A alone. (C) HUVECs were seeded on Matrigel in the presence of VEGF-A (20 ng/ml) and WMJ-S-001 (10 μM) with or without NSC-87877 at indicated concentrations. Cells were then photographed under phase-contrast after 16 h. Bar graphs show compiled data of average sprout arch numbers (n=5). *p < 0.05, compared with the control group; #*p* < 0.05, compared with the group treated with VEGF-A alone. (D) HUVECs were treated with 10 μM WMJ-S-001 for indicated time periods. Cells were then harvested for SHP-1 phosphatase activity assay as described in the *Materials and Methods* section. Data represent the mean ± S.E.M. of at least five independent experiments. * p < 0.05, compared to the vehicle-treated control group.

We next explored the mechanism by which WMJ-S-001 suppresses VEGF-A-induced VEGFR2 phosphorylation. It is conceivable that WMJ-S-001 activates a protein tyrosine phosphatase that dephosphorylates and inactivates VEGFR2 signaling. Several lines of evidence demonstrated that phosphorylation of VEGFR2 is negatively regulated by SHP-1 [20, 21, 38]. In addition, knockdown of SHP-1 by small interfering RNA (siRNA) promotes VEGF-A-induced cell proliferation in HUVECs and accelerates angiogenesis in a rat model [21, 38]. Thus, we investigated whether SHP-1 is involved in WMJ-S-001-induced VEGFR2 dephosphorylation in VEGF-A-stimulated HUVECs. As shown in Fig. 4A, NSC-87877, a SHP-1 inhibitor restored VEGFR2 Tyr1175 and Tyr1214 phosphorylation in VEGF-A-stimulated HUVECs despite the presence of WMJ-S-001. We next determined whether WMJ-S-001's suppression of cell proliferation is altered by NSC-87877 in HUVECs exposed to VEGF-A. As shown in Fig. 4B, NSC-87877 significantly restored WMJ-S-001-decreased cell proliferation in VEGF-A-stimulated HUVECs. WMJ-S-001's suppression of VEGF-A-induced tube formation was also mitigated by NSC-87877 in HUVECs (Fig. 4C). Furthermore, WMJ-S-001 caused an increase in SHP-1 phosphatase activity in a time-dependent manner (Fig. 4D). These results suggest that SHP-1 is responsible for WMJ-S-001-decreased VEGFR2 phosphorylation and subsequent angiogenesis in HUVECs exposed to VEGF-A.

### WMJ-S-001 affects cell cycle distribution and induces cell apoptosis in HUVECs

**Fig.5:**
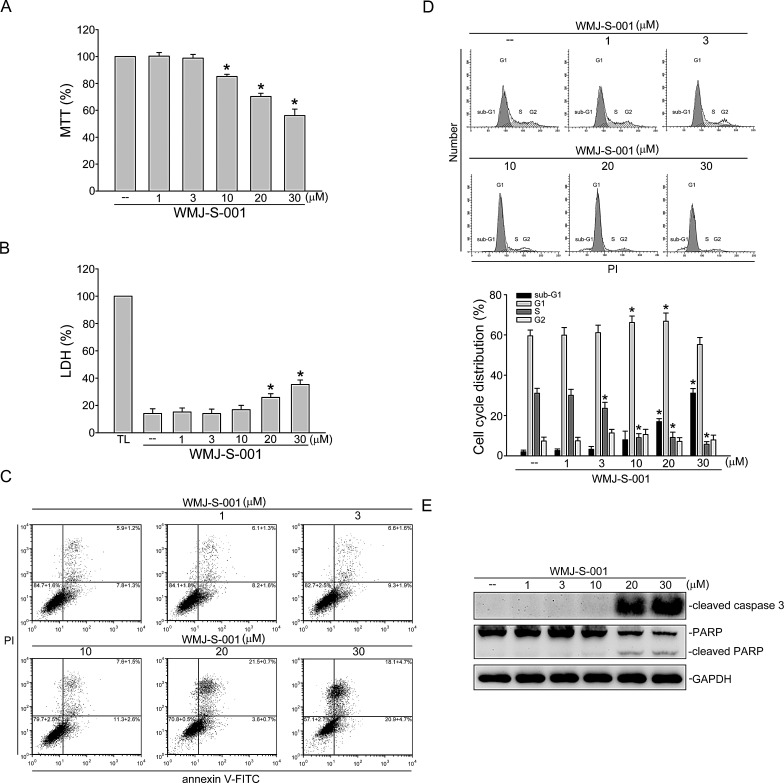
WMJ-S-001 affected cell cycle distribution and induced apoptosis in HUVECs (A) HUVECs were treated with indicated concentrations of WMJ-S-001 for 24 h. Cell viability was determined by MTT assay. Each column represents the mean ± S.E.M. of five independent experiments performed in triplicate (*p < 0.05, compared with the control group). (B) HUVECs were treated with indicated concentrations of WMJ-S-001 for 24 h. The cytotoxicity of WMJ-S-001 was determined by LDH assay. Cells were also treated with cell lysis buffer (total lysis, TL) to serve as positive control. Each column represents the mean ± S.E.M. of five independent experiments performed in triplicate (*p < 0.05, compared with the control group). (C) Cells were treated with vehicle or WMJ-S-001 at indicated concentrations for 24 h. Cells were then stained with annexin V-FITC and propidium iodide (PI) for 15 min. The percentage of apoptotic cells was then analyzed by flow-cytometric analysis. Results shown are representative of four independent experiments. (D) Cells were treated as in (C), the percentage of cells in subG1, G0/G1, S, and G2/M phases was then analyzed by flow-cytometric analysis. Each column represents the mean ± S.E.M. of four independent experiments * p < 0.05, compared with the control group (E) Cells were treated as in (C), the extent of cleavage caspase 3and PARP were then determined by immunoblotting. Results shown are representative of four independent experiments.

To determine whether WMJ-S-001 affects cell viability in HUVECs, a MTT assay was employed. As shown in Fig.5A, WMJ-S-001 concentration-dependently decreased cell viability. We next use a LDH assay to determine if the actions of WMJ-S-001 in decreasing HUVEC viability were attributable to its cytotoxic effect As shown in Fig. 5B, treatment of cells with WMJ-S-001 at 1 to 10 μM for 24 h did not significantly increase LDH release. However, WMJ-S-001 at concentrations higher than 10 μM (20 and 30 μM) significantly increased LDH release (Fig. 5B). We thus used flow-cytometric analysis with propidium iodide (PI) and annexin V-FITC labeling to detect apoptosis in HUVECs exposed to WMJ-S-001. As shown in Fig. 5C, WMJ-S-001 at 30 μM significantly increased the percentage of the annexin V^+^PI^−^ cells (lower right quadrant, early apoptotic cells) and the annexin V^+^PI^+^ cells (upper right quadrant, advanced apoptotic cells and/or necrotic cells), but concentrations below 10 μM had little effects. We also used flow-cytometric analysis with PI labeling to determine whether WMJ-S-001 affects cell cycle progression and induces apoptosis. As shown in Fig. 5D, the percentage of PI-stained cells in the S region was significantly decreased after 24 h treatment of WMJ-S-001 compared with the vehicle-treated group. These effects were accompanied by the increase in the percentage of PI-stained cells in the G1 region after exposure to WMJ-S-001 at concentrations ranging from 1 to 10 μM (Fig. 5D). In addition, WMJ-S-001 at concentrations at 10 μM or lower did not significantly induce cell apoptosis while WMJ-S-001 at concentrations higher than 10 μM (20 and 30 μM) significantly increased the percentage of PI-stained cells in the sub-G1 region (apoptotic region) (Fig. 5D). We next determined whether WMJ-S-001 activates caspase 3, which has been documented as an apoptotic marker. A selective caspase 3 substrate, PARP, was also used to confirm whether WMJ-S-001-activated caspase 3 results in PARP cleavage. As shown in Fig. 5E, WMJ-S-001 at 20 and 30 μM markedly increased cleaved form (active form) of caspase 3. These effects were accompanied by the cleavage of PARP (Fig. 5E). These results suggest that suppression of cell proliferation and induction of apoptosis may also contribute to the anti-angiogenic actions of WMJ-S-001.

### WMJ-S-001 induces p53 phosphorylation and acetylation in HUVECs

**Fig.6:**
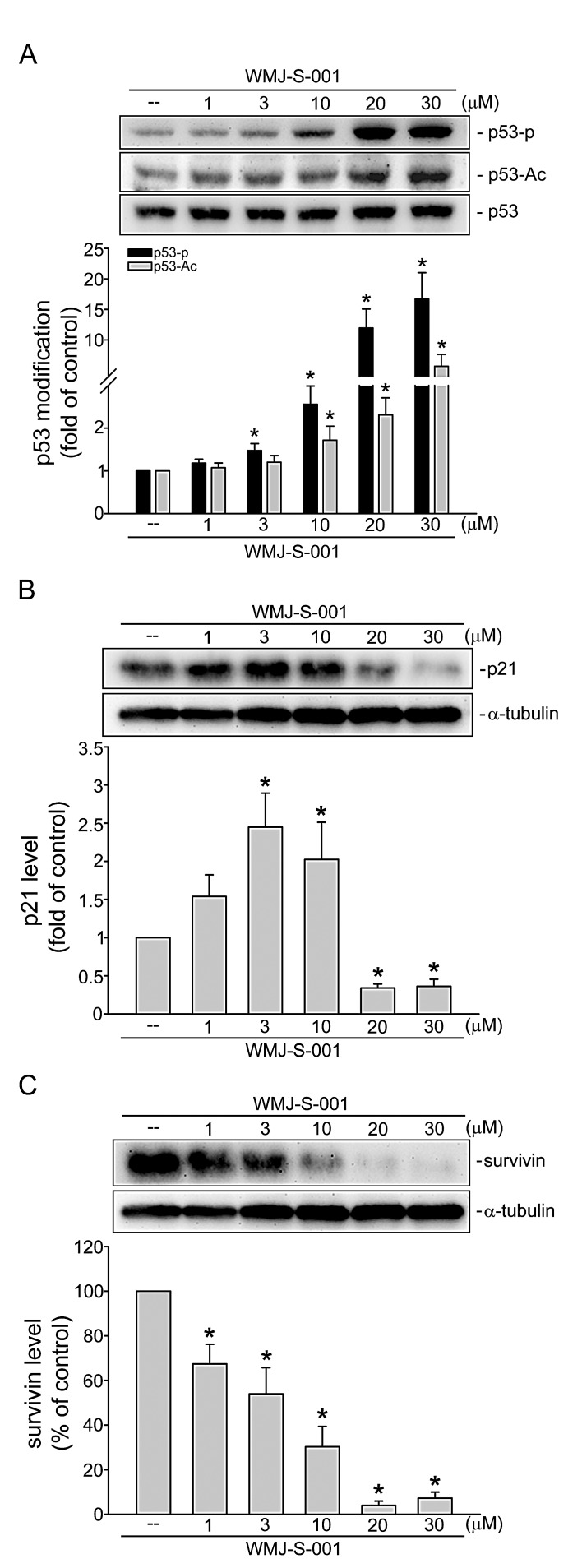
WMJ-S-001 altered p53 modification and the protein levels of p21^cip/Waf1^ and survivin in HUVECs Cells were treated with vehicle or WMJ-S-001 at indicated concentrations for 6 h. The phosphorylation and acetylation status of p53 was then determined by immunoblotting. Complied results are shown at the bottom of the chart. Each column represents the mean ± S.E.M. of four independent experiments * p < 0.05, compared with the control group. Cells were treated with vehicle or WMJ-S-001 at indicated concentrations for 24 h, the protein levels of p21 (B) and survivin (C) were then determined by immunoblotting. Each column represents the mean ± S.E.M. of four independent experiments * p < 0.05, compared with the control group.

Transcription factor p53 modulates the expression of target genes resulting in diverse cellular responses including cell cycle arrest and apoptosis [39, 40]. This transcription factor is activated via phosphorylation and acetylation [39, 41]. As shown in Fig. 6A, WMJ-S-001 at concentrations ranging from 3 to 30 μM significantly induced p53 phosphorylation and acetylation in HUVECs. Previous studies demonstrated that p53 transactivate p21^cip/Waf1^ to inhibit cell proliferation [42]. Meanwhile, an inhibitor of apoptosis (IAP) family member survivin plays a crucial role in regulating cell cycle progression and apoptosis [43, 44]. We previously demonstrated that p53 negatively regulates survivin expression [6]. Recent study showed that survivin level was elevated during angiogenesis [45]. Therefore, we examined whether WMJ-S-001 affects the levels of p21^cip/Waf1^ and survivin in HUVECs. As shown in Fig. 6B, WMJ-S-001 at 1-10 μM increased p21^cip/Waf1^ expression. In contrast, WMJ-S-001 at 20 and 30 μM decreased the level of p21^cip/Waf1^ in HUVECs (Fig. 6B). The results also demonstrated that treatment of WMJ-S-001 concentration-dependently suppressed survivin expression (Fig. 6C). These results suggest that WMJ-S-001 affects HUVEC proliferation and apoptosis through p53 activation and alterations in the protein levels of p21^cip/Waf1^and survivin.

## DISCUSSION

Cancer is one of the leading causes of death worldwide with increasing incidence. Angiogenesis plays an important role in tumor growth and metastasis [46, 47]. The inhibition of angiogenesis thus represents a potential therapeutic strategy for cancer treatment. Several angiogenesis inhibitors have already shown benefit in suppressing tumor growth and metastasis in various types of solid tumors [2, 48]. These findings led to increased efforts to discover and develop novel angiogenesis inhibitors. There is growing evidence of the beneficial effects of hydroxamate derivatives in the treatment of cancer [28, 31, 32]. In this study, we have identified an aliphatic hydroxamate derivative, WMJ-S-001, which suppressed both VEGF-A-induced angiogenesis ex vivo and tumor-induced angiogenesis *in vivo*. We demonstrated that WMJ-S-001 activates SHP-1 to inhibit VEGFR2 signaling and subsequent angiogenesis. p53 signaling and alterations of p21 and survivin levels may also contribute to the anti-angiogenic effects of WMJ-S-001. Our results also showed that suppressing angiogenesis may contribute to WMJ-S-001 reduction of tumor growth in a tumor xenograft model.

VEGF-A-VEGFR2 signaling plays a pivotal role in angiogenesis. It appears that suppression of VEGFR2 signaling is a promising anti-angiogenic strategy for cancer treatment [49, 50]. Phosphorylation of VEGFR2 residues is required for the activation of its downstream signaling pathways including Src, Akt, ERK and FAK, which regulate endothelial cell survival, proliferation and motility [16, 25]. There are several VEGFR2 tyrosine residues that become phosphorylated after VEGF-A exposure. Among these residues, Tyr1175 is believed to be the most important in angiogenic signaling in VEGF-A-stimulated HUVECs [51]. Our results demonstrated that WMJ-S-001 inhibited the phosphorylation of not only VEGFR2 Tyr1175, but also Tyr1214 and in downstream kinases such as Src, Akt, ERK and FAK in HUVECs exposed to VEGF-A. Ultimately, WMJ-S-001 attenuated angiogenesis in both ex vivo and *in vivo* animal models using VEGF-A- and HCT116 colorectal cancer cells as stimuli, respectively. In summary, the inhibition of VEGFR2 and subsequent signaling cascades may be responsible for WMJ-S-001's anti-angiogenic effects.

The mechanisms that regulate VEGFR2 phosphorylation remain incompletely understood. There are many protein tyrosine phosphatases (PTPs) that regulate growth factor effects in endothelial cells [52]. Among these PTPs, SHP-1 has been reported to negatively regulate VEGFR2 phosphorylation. Sugano et al [21] also demonstrated that knockdown of SHP-1 by siRNA accelerates angiogenesis in a rat model. Consistent with those observations, we noted that WMJ-S-001-inhibited cell proliferation and tube formation were causally related to SHP-1 activation. We also noted that SHP-1 inhibitor reversed WMJ-S-001's dephosphorylation of VEGFR2 in VEGF-A-stimulated HUVECs. These findings suggest that SHP-1 plays a pivotal role in VEGFR2 dephosphorylation and subsequent signaling events in the presence of WMJ-S-001. Moreover, VEGFR2 phosphorylation is reported to be regulated by density enhanced phosphatase (DEP)-1, VE-PTP and PTP1B. DEP-1 [53] or VE-PTP [54] deletion leads to increased VEGFR2 phosphorylation, and consequent activation of downstream signaling cascades. PTP1B was shown to negatively regulate VEGF-A signaling by dephosphorylating VEGFR2 [55]. It raises the possibility that PTPs other than SHP-1 contribute to WMJ-S-001 dephosphorylation of VEGFR2. Further investigations are needed to characterize whether other PTPs such as DEP-1, VE-PTP, or PTP1B contribute to the anti-angiogenic actions of WMJ-S-001.

We also demonstrated that WMJ-S-001 induces the activation of p53 and its downstream target, p21, the cell cycle regulator. The elevated levels of p21 by WMJ-S-001 provide a barrier to proliferation by blocking cell cycle machinery. It appears additional cycle regulatory proteins contribute to the anti-proliferative actions of WMJ-S-001. While WMJ-S-001 at a concentration of ≤ 10 μM inhibited cell cycle progression, it is capable of inducing caspase 3 activation and cell apoptosis at a concentration of >10 μM. Similar to the previous report that caspase 3-mediated cleavage of p21 convers cells from growth arrest to undergo apoptosis [56], we also noted that WMJ-S-001-activated caspase 3 is accompanied by a decrease in p21 level. On the other hand, it is reported that survivin level was found elevated during angiogenesis [45]. Survivin plays a critical role in regulating not only apoptosis, but also mitosis [57]. We have previously shown that p53 negatively regulated survivin expression [39]. Therefore, p53-mediated down-regulation of survivin may also contribute to the anti-proliferative, apoptotic and anti-angiogenic actions of WMJ-S-001 in HUVECs. The molecular mechanism underlying WMJ-S-001 activation of p53 has not been delineated. We recently found that SHP-1 inhibitor NSC-87877 restored the WMJ-S-001's effects on p21 and survivin levels in HUVECs (unpublished data). Chen et al [58] further demonstrated that activation of SHP-1-PP2A-p38MAPK cascade induces p53 phosphorylation and activation in vascular smooth muscle cells. Taken together, these findings suggest that SHP-1 mediates WMJ-S-001-induced p53 activation and alterations of p21 and survivin. However, further investigations are needed to clarify the causal role of SHP-1 and the underlying mechanisms of WMJ-S-001-induced p53 activation in HUVECs.

In addition to targeting VEGFR2 signaling, apoptotic mechanisms may also contribute to WMJ-S-001's anti-angiogenic effects when its concentration is higher than 10 μM. Moreover, we recently noted that WMJ-S-001 not only suppresses angiogenesis, but also inhibits cell proliferation and induces apoptosis in HCT116 colorectal cancer cells (unpublished data). It suggests that anti-proliferative and apoptotic effects of WMJ-S-001 on tumor cells may also contribute to its anti-tumor actions. The different mechanisms of WMJ-S-001's actions in suppressing angiogenesis and tumor cell proliferation remain to be elucidated. It is likely that targeting angiogenesis and tumor cell proliferation culminates in the suppression of tumor growth.

In summary, we have demonstrated in this study that WMJ-S-001 exhibits anti-angiogenic effects through at least two signaling pathways: SHP-1-mediated suppression of VEGF-A-VEGFR2 signaling and p53-mediated alterations of p21 and survivin in HUVECs. These results support the potential of WMJ-S-001 as a valuable lead compound in the development of anti-angiogenic agent in future oncologic therapy.

## Materials and methods

### Reagents

3-[4, 5-dimethylthiazol-2-yl]-2, 5-diphenyltetrazolium bromide (MTT), toluidine blue O, and McCoy5A medium were from Sigma-Aldrich (St Louis, MO, USA). Medium 199 (M199), fetal bovine serum (FBS), and all cell culture reagents were purchased from Invitrogen (Carlsbad, CA, USA). NSC-87877 was purchased from Millipore (Billerica, MA, USA). Antibodies against VEGFR2, VEGFR2 phosphorylated at tyrosine 1175 (Y1175), ERK1/2, ERK1/2 phosphorylated at threonine 202/tyrosine 204 (T202/Y204), Akt, Akt phosphorylated at serine 473 (S473), FAK and FAK phosphorylated at tyrosine 397 (Y397), Src and Src phosphorylated at tyrosine 416 (Y416), p53 phosphorylated at serine 15 (S15) and p53 acetylated at lysine 379 (K379), caspase 3 active form and PARP were purchased from Cell Signaling (Danvers, MA, USA). Antibodies against α-tubulin and VEGFR2 phosphorylated at tyrosine 1214 (Y1214) were purchased from Novus (Littleton, CO, USA). Antibodies specific for SHP-1, p21 and p53 were purchased from Santa Cruz Biotechnology (Santa Cruz, CA, USA). Antibodies against survivin and anti-mouse and anti-rabbit IgG conjugated peroxidase antibodies were purchased from GeneTex Inc (Irvine, CA, USA). The enhanced chemiluminescence detection kit was from GE Healthcare (Little Chalfont, UK). Cell Proliferation ELISA, BrdU assay kit was acquired from Roche (Indianapolis, IN, USA). All materials for immunoblotting were purchased from GE Healthcare (Little Chalfont, UK). All other chemicals were obtained from Sigma-Aldrich (St. Louis, MO, USA).

### Synthesis of WMJ-S

50% NH_2_OH_(aq)_ (2 mL) was added to a solution of benzoyl lovastatin (501 mg, 1 mmol) in THF (3 mL), which was prepared according to the method reported by Dabak and colleagues. This solution was stirred at room temperature for 3 h. The reaction mixture was diluted with distilled H_2_O (50 mL), acidified with 1N HCl_(aq)_ to pH 4-5, and extracted with EtOAc (25 mL x 3). The organic layer was dried over Na_2_SO_4_, filtered and the solvent concentrated in vacuo. The residue was purified by silica gel chromatography (EtOAc: *n*-hexane=1: 1~2: 1) to give WMJ-S (374 mg, 69%) as a sold. mp 70-75 °C (acetone); IR (KBr) *ν_max_* cm^−1^ 3902, 3853, 3838, 3749, 3735, 3648, 3566, 2965, 1716, 1683, 1625, 1558, 1540, 1507, 1456; ^1^H NMR (500 MHz, CDCl_3_) δ 9.73 (1H, s), 7.98 (2H, d, *J* = 7.6 Hz), 7.53 (1H, t, *J* = 7.2 Hz), 7.40 (2H, t, *J* = 7.6 Hz), 5.95 (1H, d, *J* = 9.7 Hz), 5.72 (1H, dd, *J* = 6.2, 9.4 Hz), 5.53 (1H, t, *J* = 5.9 Hz), 5.49 (1H, s), 5.37 (1H, d, *J* = 2.2 Hz), 3.80 (1H, s), 2.68 (2H, dd, *J* = 6.8, 14.0 Hz), 2.42 (1H, m), 2.29 (2H, dd, *J* = 6.9, 13.8 Hz), 2.20 (1H, m), 1.94 (2H, m), 1.88 (2H, m), 1.60 (3H, m), 1.46 (1H, m), 1.36 (1H, m), 1.30 (1H, m), 1.25 (1H, t, *J* = 7.2 Hz), 1.19 (1H, m), 1.10 (1H, m), 1.05 (3H, d, *J* = 5.5 Hz), 1.04 (3H, d, *J* = 6.6 Hz), 0.87 (1H, m), 0.84 (3H, d, *J* = 6.9 Hz), 0.79 (3H, t, *J* = 7.4 Hz); ^13^C NMR (125 MHz, CDCl_3_) δ 178.3, 168.9, 166.8, 134.2, 134.0, 132.0, 130.8, 130.5, 130.2, 129.2, 129.0, 70.9, 69.5, 69.1, 61.2, 42.4, 38.3, 38.2, 37.3, 35.6, 33.6, 31.4, 28.5, 27.6, 25.2, 23.7, 17.0, 14.7, 12.4; HRESIMS *m/z* 542.3151 [M+H]^+^ (caclcd for C_31_H_44_NO_7_, 542.3118).

### Ethic statement

This study was carried out in strict accordance with the recommendations in the Guide for the Care and Use of Laboratory Animals of the National Institutes of Health. The protocols described below were approved by the Taipei Medical University Laboratory Animal Care and Use Committee (Permit Number: LAC-100-0097). All surgery was performed under sodium pentobarbital anesthesia, and all efforts were made to minimize suffering.

### Cell culture

Human umbilical vascular endothelial cells (HUVECs) and HCT116 colorectal cancer cell line were obtained from the Bioresource Collection and Research Center (Hsinchu, Taiwan). HUVECs were maintained in M199 medium containing vascular endothelial cell growth supplement (ECGS) (Millipore), 10% FBS, 5 U/ml heparin, 20 mM HEPES, 100 U/ml of penicillin G, and 100 μg/ml streptomycin in a humidified 37 °C incubator. HCT116 cells were maintained in McCoy5A containing 10% FBS, 100 U/ml of penicillin G, and 100 μg/ml streptomycin in a humidified 37 °C incubator.

### Cell viability assay (MTT assay)

Cell viability was measured by the colorimetric 3-(4,5-dimethylthiazol-2-yl)-2,5-diphenyl tetrazolium bromide (MTT) assay as described previously [39].

### Lactate dehydrogenase (LDH) release assay

LDH leakage was measured to quantify cytotoxicity with a CytoTox96 non-radioactive cytotoxicity assay kit (Promega) as described previously [59].

### Cell proliferation assay (BrdU incorporation assay)

HUVECs (2×10^4^ per well) were seeded in 96-well tissue culture plates and incubated for 24 h. Cells were then starved in M199 medium containing 2% FBS in the absence of endothelial cell growth supplements for another 16 h. After starvation, cells were pre-treated for 30 min with various concentrations of WMJ-S-001, followed by the stimulation with VEGF (20 ng/ml) for another 24 h. Cell proliferation was then determined using a Cell Proliferation ELISA, BrdU (colorimetric) kit (Roche) based on the colorimetric detection of the incorporation of BrdU, following the manufacturer's instructions.

### Flow cytometric analysis

HUVECs were incubated with WMJ-S-001 at indicated concentrations for 24 h. At the end of the experiments, cells were washed twice with PBS and re-suspended in ice-cold 70% ethanol at 0 °C overnight. Cells were washed with phosphate-citric acid buffer and subsequently stained with propidium iodide (PI) staining buffer containing 0.1% Triton X-100, 100 μg/ml RNase A, and 40 μg/ml PI for 30 min in the dark. Cells were then filtered on a nylon mesh filter. The samples were analyzed using the FACScan and Cellquest program (BD Biosciences, San Jose, CA. USA). The ModFit programs (BD Biosciences, San Jose, CA) were used to determine the percentage of PI-stained cells in the subG1 (Apoptosis, Apo), G0/G1, S or G2/M region. On the other hand, apoptotic cells were also detected by propidium iodide (PI) and annexin V-FITC labeling as described previously [60]. The double labeling was performed at 37 °C by treating cells with PI (50 μg/ml) and annexin V-FITC (2 μg/ml) for 15 min in the dark. The staining was then immediately analyzed using the FACScan and Cellquest program. The FCS Express program (BD Biosciences, San Jose, CA) was used to determine the percentage of stained cells in different quadrants. The lower left quadrant of each panel (annexin V^−^PI^−^) shows the viable cells, which exclude PI and are negative for annexin V binding. The lower right quadrant (annexin V^+^PI^−^) represents the early apoptotic cells, annexin V positive and PI negative, demonstrating cytoplasmic membrane integrity. The upper right quadrant (annexin V^+^PI^+^) contains advanced apoptotic cells and necrotic cells, which are positive for annexin V binding and for PI uptake.

### Transwell invasion assay

Transwell invasion assay was done as described previously [59]. Briefly, the bottom face of the filter in the transwell plate (Corning, NY, USA) was coated with 0.2 % gelatin. The bottom chambers were filled with M199 medium containing 2 % FBS in the presence of VEGF-A (20 ng/ml) and HUVECs (10^4^ cells per well) in 200 μL M199 medium (without growth factors) were seeded in the top chambers. The top chamber contained vehicle or various concentrations of WMJ-S-001. Cells were allowed to invade for 16 h. Non-invaded cells (on the top side of filter) were scraped with a cotton swab, and invaded cells were fixed and stained with 0.5% toluidine blue in 4% paraformaldehyde. The cells were photographed and quantified by counting the number of stained cells under an inverted contrast phase microscope (Nikon, Japan).

### Matrigel tube formation assay

The tube formation assay was performed as described previously [59]. Matrigel, a basement membrane matrix (Becton Dickinson, Mountain View, CA, USA), was polymerized at 37 °C for 30 min. HUVECs suspended in M199 medium containing 2 % FBS in the presence or absence of VEGF-A (20 ng/ml) were seeded onto the Matrigel. They were then treated with vehicle or WMJ-S-001 at indicated concentrations. After 16 h, cells were photographed using phase-contrast microscopy.

### Immunoblot analysis

Immunoblot analyses were performed as described previously [61]. Briefly, cells were lysed in an extraction buffer containing 10 mM Tris (pH 7.0), 140 mM NaCl, 2 mM PMSF, 5 mM DTT, 0.5% NP-40, 0.05 mM pepstatin A, and 0.2 mM leupeptin. Samples of equal amounts of protein were subjected to SDS-PAGE and transferred onto a NC membrane which was then incubated in a TBST buffer containing 5% non-fat milk. Proteins were visualized by incubating with specific primary antibodies followed by horseradish peroxidase-conjugated secondary antibodies. Immunoreactivity was detected based on enhanced chemiluminescence per the instructions of the manufacturer. Quantitative data were obtained using a computing densitometer with a scientific imaging system (Biospectrum AC System, UVP).

### Aortic ring sprouting assay

Assay was performed as described previously[59]. Aortic arch was dissected from 8 to 10-week-old Sprague-Dawley rats. After removing the surrounding fibro-adipose tissues and thoroughly rinsing with M199 culture medium, the aortas were cut into about 1 mm ring segments. The aortic rings were immersed in Matrigel in the wells of 48-well plate. VEGF-A (20 ng/ml) with or without WMJ-S-001 was then added to the wells. The aortic rings were cultured in 37 °C with 5% CO_2_ and the cultured medium was changed every 3 days. Growing sprouts of endothelial cells were observed and photographed on day 8. The images were photographed under microscope, and sprouting area was determined on the computer-digitized images with Image-Pro Plus (Media Cybernetics) software. The analysis of sprouting area was done by an observer who was blinded to the treatment group. All protocols were approved by the Taipei Medical University Laboratory Animal Care and Use Committee

### VEGF-A or Tumor cells-induced angiogenesis Matrigel plug assay

3-5 week old nude_nu/nu_ mice were obtained from BioLasco (Taipei, Taiwan) and housed in clean specific pathogen free (SPF) rooms. An aliquot (300 μl) of Matrigel containing VEGF (200 ng/ml) with heparin (20 U) was injected subcutaneously into the right flank of each mouse (VEGF-A-induced angiogenesis model). In the other set of experiments, HCT116 cells were harvested and re-suspended in PBS. Cells (5×10^6^ cells) in a volume of 150 μl in the presence of heparin (20 U) were mixed with Matrigel (150 μl) and then injected subcutaneously into the right flank of each mouse (Tumor cells-induced angiogenesis model). After implantation, animals were randomized to either the vehicle-treated control group or the treatment group, which received WMJ-S-001 20 mg/kg/day. The treatment was administered intraperitoneally once daily for 10 days. At the end of treatment, animals were sacrificed, Matrigel plugs were removed and the surrounding tissues trimmed. Hemoglobin levels of the Matrigel plugs were evaluated with Drabkin's reagent kit (Sigma-Aldrich) according to the manufacturer's instructions. The concentration of hemoglobin was calculated based on a set of hemoglobin standards. All protocols were approved by the Taipei Medical University Laboratory Animal Care and Use Committee

### Mouse xenograft colorectal tumor model

3-5 week old nude_nu/nu_ mice were obtained from BioLasco (Taipei, Taiwan) and housed in clean specific pathogen free (SPF) rooms. HCT116 cells were harvested and resuspended in PBS, and 5×10^6^ cells in a volume of 0.3 mL were injected subcutaneously into the right flank of each mouse. Once the tumor reached approximately 150 mm^3^, animals were randomized into the control group and the treatment groups, which received WMJ-S-001 20 mg/kg/day. The treatment was administered intraperitoneally once daily for 22 days. Tumors were measured every day by a digital caliper. Tumor volume was calculated using the formula *V* (mm^3^) = [*ab*^2^] × 0.52, where *a* is the length and *b* is the width of the tumor [39]. At the end of treatment, animals were sacrificed and tumors were removed. All protocols were approved by the Taipei Medical University Animal Care and Use Committee.

### Immunohistochemical anylysis

Multiple cryosections were obtained from HCT116 tumor xenografts. CD31^+^ vessel area was assessed using rabbit anti-mouse CD31 antibody (Abcam, Cambridge, MA, USA) and peroxidase-conjugated goat anti-rabbit antibody (The Jackson Laboratory, Sacramento, CA, USA). Antibody binding was visualized using stable diaminobenzidine. Images were obtained in four different quadrants of each tumor section at ×40 magnification. Measurement of vessel area of CD31-stained vessels was done as described previously [62].

### SHP-1 activity assay

A tyrosine phosphatase assay system (Promega, Madison, WI, USA) was used to measure phosphate release as an index of phosphatase activity according to the manufacturer's instructions with modifications. Briefly, 200 μg of cellular proteins were incubated for 2 h at 4 °C with 2 μg anti-SHP1 antibody (Santa Cruz Biotechnology, Santa Cruz, CA, USA), and 20 μl protein A-Magnetic Beads (Millipore, Billerica, MA, USA), to immunoprecipitate SHP-1. Immune complexes were then collected, washed three times, and incubated with phosphoprotein, the substrate (amino acid sequence END(pY)INASL, 100 μM), in protein phosphatase assay buffer (20 mM 4-morpholinepropanesulfonic acid (pH 7.5), 60 mM 2-mercaptoethanol, 0.1 M NaCl, and 0.1 mg/ml serum albumin). Reactions were initiated by the addition of the phosphoprotein substrate and carried out for 20 min at 30 °C. We also prepared appropriate phosphate standard solutions containing free phosphate for standard curve. Reactions were terminated by the addition of 50 μl of the Molybdate Dye solution. The absorbance at 630 nm was measured on a microplate reader. Nonspecific hydrolysis of END(pY)INASL by lysates was assessed in normal IgG immunoprecipitates.

### Statistical analysis

Results are presented as the mean ± S.E. from at least three independent experiments. One-way analysis of variance (ANOVA) was followed by the Newman-Keuls test, when appropriate, to determine the statistical significance of the difference between means. A *p* value of < 0.05 was considered statistically significant.

## Abbrevations

ERK: extracellular signal-regulated kinase
FAK: focal adhesion kinase
HUVECs: human umbilical endothelial cells
IP: intraperitoneal injections
MTT: 3-(4,5-dimethylthiazol-2-yl)-2,5-diphenyl tetrazolium bromide
PI: propidium iodide
VEGF: vascular endothelial growth factor
